# Improvement of thermoelectric properties and their correlations with electron effective mass in Cu_1.98_S_*x*_Se_1−*x*_

**DOI:** 10.1038/srep40436

**Published:** 2017-01-16

**Authors:** Lanling Zhao, Frank Yun Fei, Jun Wang, Funing Wang, Chunlei Wang, Jichao Li, Jiyang Wang, Zhenxiang Cheng, Shixue Dou, Xiaolin Wang

**Affiliations:** 1 School of Physics, Shandong University, Jinan, 250100, P.R. China; 2 Spintronic and Electronic Materials Group, Institute for Superconducting and Electronic Materials, Australian Institute for Innovative Materials, University of Wollongong, North Wollongong, 2500, Australia; 3 Key Laboratory for Liquid-Solid Structural Evolution and Processing of Materials, Shandong University, Jinan, 250061, China; 4 Institute for Crystal Materials, Shandong University, Jinan, 250100, P. R. China

## Abstract

Sulphur doping effects on the crystal structures, thermoelectric properties, density-of-states, and effective mass in Cu_1.98_S_*x*_Se_1−*x*_ were studied based on the electrical and thermal transport property measurements, and first-principles calculations. The X-ray diffraction patterns and Rietveld refinements indicate that room temperature Cu_1.98_S_*x*_Se_1−*x*_ (x = 0, 0.02, 0.08, 0.16) and Cu_1.98_S_*x*_Se_1−*x*_ (x = 0.8, 0.9, 1.0) have the same crystal structure as monoclinic-Cu_2_Se and orthorhombic-Cu_2_S, respectively. Sulphur doping can greatly enhance *zT* values when *x* is in the range of 0.8≤ × ≤1.0. Furthermore, all doped samples show stable thermoelectric compatibility factors over a broad temperature range from 700 to 1000 K, which could greatly benefit their practical applications. First-principles calculations indicate that both the electron density-of-sates and the effective mass for all the compounds exhibit non-monotonic sulphur doping dependence. It is concluded that the overall thermoelectric performance of the Cu_1.98_S_*x*_Se_1−*x*_ system is mainly correlated with the electron effective mass and the density-of-states.

The dimensionless thermoelectric figure-of-merit (*zT*) is defined as *zT* = *S*^2^*σ*T/*κ* = S^2^*σ*T/(*κ*_c_ + *κ*_L_), where *S*, T, *σ, κ, κ*_c_, and *κ*_L_ are the Seebeck coefficient, absolute temperature in Kelvin, electrical conductivity, total thermal conductivity, charge carrier thermal conductivity, and lattice thermal conductivity, respectively^1^,^2^,^3^,^4^. Enhanced *zT* values could be realized through adjusting the electronic structures and thermal conductivity by the doping approach^5^,^6^,^7^,^8^,^9^. It should be noted that *zT* is proportional to the square of *S*, indicating that improving *S* might be an easier way to get improved *zT* values, compared to regulating the other thermoelectric parameters such as *σ* and *κ*.

It is well known that *σ* and *S* can be estimated by the following formulas^10^,^11^,^12^,


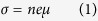



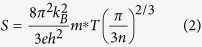


where *n, μ, k*_*B*_, *h*, and m^*^ are the charge carrier density, carrier mobility, Boltzmann constant, Planck constant and effective mass of the carriers, respectively. Moreover, the effective mass (m*) and carrier mobility (*μ*) can be deduced from the electronic band structures and density-of-states (DOS) obtained from related first-principle calculations^13^. In this regard, *S* can be predicted through performing theoretical calculations on the electronic band structures and DOS, and the estimation of *σ* can also be roughly achieved with considering the average scattering time as a constant^14^,^15^. Therefore, it is highly desirable to gain insight into the electronic structures to get theoretical backup for the observed experimental phenomena.

Among all the state-of-the-art high temperature *p*-type thermoelectric materials, the copper-ion-liquid-like Cu_2−*x*_Se and Cu_2−*x*_S compounds show high thermoelectric performance, even though there still are some issues that need to be resolved before practical applications can be considered^16^. It has been reported that polycrystalline Cu_2−*x*_Se and Cu_2−*x*_S bulks can achieve the highest *zT* values of around 1.6 and 1.7 at 1000 K^17^,^18^, which have been further improved to 1.8 and 1.9^19^,^20^, respectively.

Additionally, it should be noted that the high-temperature *β*-Cu_2−*x*_Se and *α*-Cu_2−*x*_S have the same crystal structure, and both of them are superionic conductors. Besides the high crystal symmetry, these two systems also possess another important property, that of congruent melting, which means that highly dense samples can be easily fabricated by a facile melt-solidification technique.

The previous studies^20^,^21^,^22^ on the electronic structures for Cu_2_Se and Cu_2_S compounds indicate that the copper deficiency makes both of them intrinsic *p*-type conductors owing to the contributions mainly from the Cu 3d-, Se 4p-, and S 3p-states near the Fermi level (*E*_F_). These facts provide evidence that doping other elements into Cu or Se (S) sites could effectively alter their electronic structures, and consequently affect their electrical and thermal transport behaviour as well as their overall thermoelectric performance.

Generally, in order to obtain enhanced *S* and concurrent high *σ* as well as low *κ* values through the doping approach, the following factors should be considered when choosing dopants: (1) Dopants should have the same valence as the counterpart element, which will ensure the charge balance of the system and maintain the same crystal structure. (2) Dopants should have comparable radiuses to the counterpart element, which will result in little difference in the lattice parameters and provide good optimization of electrical and thermal transport properties.

As for the Cu_2−*x*_Se system, the doping approach, using such elements as Ag, Sb, Al, and Sn for the Cu sites^23^,^24^,^25^,^26^,^27^,^28^ and Te and I for the Se sites^21^,^29^, has been chosen to modify its electronic structures and thermal conductivity up to now. The results illustrate, however, that only a small amount doping with one of these elements could lead to limited improvements to the thermoelectric performance in this system. Generally, the lighter atoms and heavier atoms should have opposite effects on the electronic and thermal transport properties. For the substitutions on Se sites, only the heavier atoms have been investigated so far, and no enhanced thermoelectric performance was observed except at the phase transition temperatures. Therefore, it is necessary to investigate the doping effects of lighter atoms, with a smaller atomic radius, to test whether or not the doping approach could be beneficial for the further enhancement of the overall thermoelectric performance of the Cu_2−*x*_Se system.

In this work, we investigated sulphur doping effects on the thermoelectric properties of the Cu_2−*x*_Se system based on the following considerations: (1) At high temperature, *α*-Cu_2_S has the same crystal structure as *β*-Cu_2_Se, and therefore, sulphur should be very easy to substitute into the lattice and replace Se. (2) S^2−^ has the same valence as Se^2−^, which should result in good electron balance in this system. (3) The unit cell size should be reduced after S substitutes for Se, which will affect the migration of copper ions and lead to varied electrical and thermal transport behaviour.

It should be noted that, for practical applications of thermoelectric materials, besides the high *zT* values, the thermoelectric compatibility factor (s), derived as 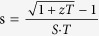, is another important factor, which is crucial for the efficient operation of a high temperature thermoelectric device^12^,^30^,^31^,^32^,^33^. The closer the s for two *n*- and *p*-type thermoelectric materials, the higher the combined efficiency that will be achieved when they are adjoining segments in one thermoelectric device. Little information on s, however, has been reported for the Cu_2−*x*_Se or Cu_2−*x*_S based thermoelectric materials. Hence, it is meaningful to calculate the s values for the sulphur doped Cu_2−*x*_Se to gain sufficient information for their future practical applications.

Herein, the doping effects on the thermoelectric properties of highly dense Cu_1.98_S_*x*_Se_1−*x*_ polycrystalline bulks were investigated experimentally, in order to provide a full understanding of how the doping approach modifies the thermoelectric properties of the Cu_2−*x*_Se system. We also conducted a systematic study on the sulphur doping effects on the electronic band structures and DOS for the Cu_15_S_*x*_Se_8−*x*_ compounds based on Density Functional Theory (DFT) calculations. The results indicate that the overall thermoelectric performance in Cu_1.98_S_*x*_Se_1−*x*_ is strongly dependent on the sulphur doping concentration, and it is mainly correlated with the electron effective mass and DOS.

## Results and Discussion

**Figure 1:**
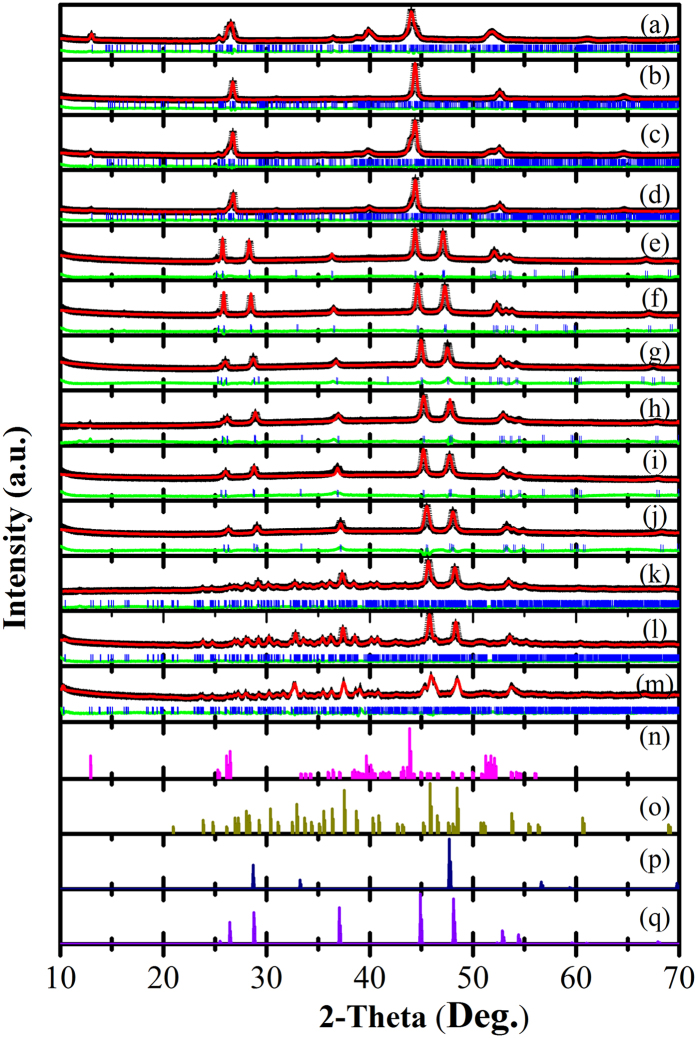
X-ray diffraction (XRD) patterns of the fabricated Cu_1.98_S_*x*_Se_1−*x*_ (*x* = 0, 0.02, 0.08, 0.16, 0.2, 0.3, 0.4, 0.5, 0.6, 0.7, 0.8, 0.9, 1.0) samples: (**a**) Cu_1.98_Se, (**b**) Cu_1.98_S_0.02_Se_0.98_, (**c**) Cu_1.98_S_0.08_Se_0.92_, (**d**) Cu_1.98_S_0.16_Se_0.84_, (**e**) Cu_1.98_S_0.2_Se_0.8_, (**f**) Cu_1.98_S_0.3_Se_0.7_, (**g**) Cu_1.98_S_0.4_Se_0.6_, (**h**) Cu_1.98_S_0.5_Se_0.5_, (**i**) Cu_1.98_S_0.6_Se_0.4_, (j) Cu_1.98_S_0.7_Se_0.3_, (**k**) Cu_1.98_S_0.8_Se_0.2_, (**l**) Cu_1.98_S_0.9_Se_0.1_, (**m**) Cu_1.98_S, (**n**) standard XRD pattern for monoclinic structured Cu_2_Se, (**o**) standard XRD pattern for orthorhombic structured Cu_2_S, (**p**) standard XRD pattern for cubic structured Cu_1.8_Se, and (**q**) standard XRD pattern for hexagonal structured Cu_2.001_S. (**+** data points, − calculation line, − difference line, **|** marker points).

Figure 1 shows the X-ray diffraction (XRD) patterns for the fabricated Cu_1.98_S_*x*_Se_1−*x*_ (*x* = 0, 0.02, 0.08, 0.16, 0.2, 0.3, 0.4, 0.5, 0.6, 0.7, 0.8, 0.9, 1.0) samples. The results indicate that the Cu_1.98_S_*x*_Se_1−*x*_ samples show different crystal structures with different *x* values. They are single-phase and have the same monoclinic^34^ crystal structure as the low temperature *α*-phase Cu_2−*x*_Se (PDF No.: 27–1131^23^) when *x* ≤ 0.16. They then become composites of low temperature cubic structured Cu_1.8_Se and hexagonal structured Cu_2.001_S when *x* varies in the range from 0.2 to 0.7 (0.2 ≤ *x* ≤ 0.7). Finally, they become single-phase orthorhombic structured Cu_2_S (PDF: 23–961) when *x* is over 0.8.

**Figure 2:**
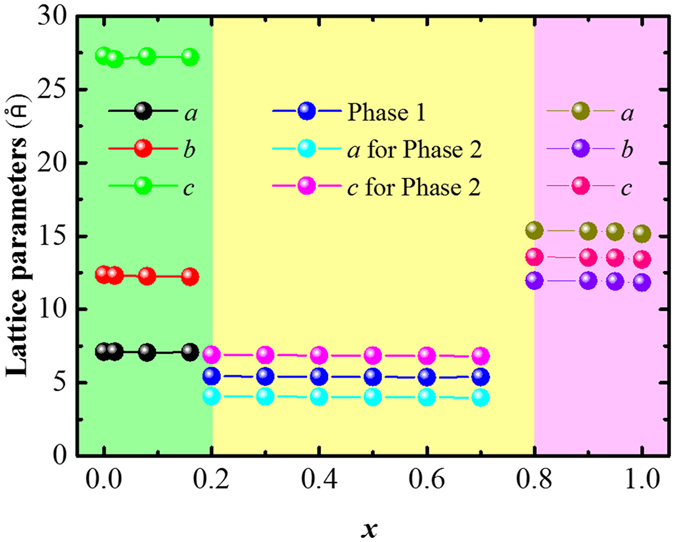
Phase diagram of sulphur doping level dependence of the crystal structures at room temperature. (I: monoclinic structured Cu_2_Se phase; II: Composites of cubic structured Cu_1.8_Se (Phase 1) and hexagonal structured Cu_2.001_S (Phase 2); III: orthorhombic structured Cu_2_S phase).

Rietveld refinements were performed for all samples based on the obtained X-ray diffraction patterns and the deduced lattice parameters, and the R-factors are listed in Table S1 (Supplementary Information). The summarized phase diagram for the sulphur doping level dependence of the lattice parameters is also displayed in Fig. 2, revealing that the Cu_1.98_S_*x*_Se_1−*x*_ (*x* = 0.02, 0.08, 0.16) samples have slightly reduced lattice parameters in comparison with the Cu_1.98_Se sample, due to the smaller radius of sulphur compared to that of selenium. In the same way, the Cu_1.98_S_0.8_Se_0.2_ and Cu_1.98_S_0.9_Se_0.1_ samples have enlarged lattice parameters in contrast to the Cu_1.98_S sample, owing to the larger radius of selenium.

The geometry optimized primitive cells for the Cu_15_S_*x*_Se_8−*x*_ (*x* = 0, 1, 2, 4, 6, 8) compounds are displayed in Figure S2, and the lattice parameters for these compounds are summarized in Table S1 which exhibit the consistent variation trend as the obtained experimental results. The Cu_15_Se_8_ has the largest volume of 387.606 Å^3^, while the Cu_15_S_8_ has the smallest volume of 340.486 Å^3^. The Cu_15_S_*x*_Se_8−*x*_ (*x* = 1, 2, 4, 6) compounds have the volume values between 352.784 and 378.266 with values decreased with increasing *x*.

Since both Cu_2−*x*_Se and Cu_2−*x*_S are superionic conductors, and the migration of copper ions plays important roles on the system’s electrical conductivity, the changed lattice parameters should lead to different electrical conductivity and Seebeck coefficient, which will, in turn, result in modified thermoelectric properties with different *zT* values. Thus, it is essential to discuss the sulphur doping effects on the thermoelectric properties of the Cu_2−*x*_Se system, as well as the selenium doping effects on the thermoelectric properties of the Cu_2−*x*_S system. Hence, we will focus on the thermoelectric properties of the single-phase Cu_1.98_S_*x*_Se_1−*x*_ (*x* = 0.02, 0.08, 0.16) and Cu_1.98_S_*x*_Se_1−*x*_ (*x* = 0.8, 0.9, 1.0) samples in the following part.

**Figure 3:**
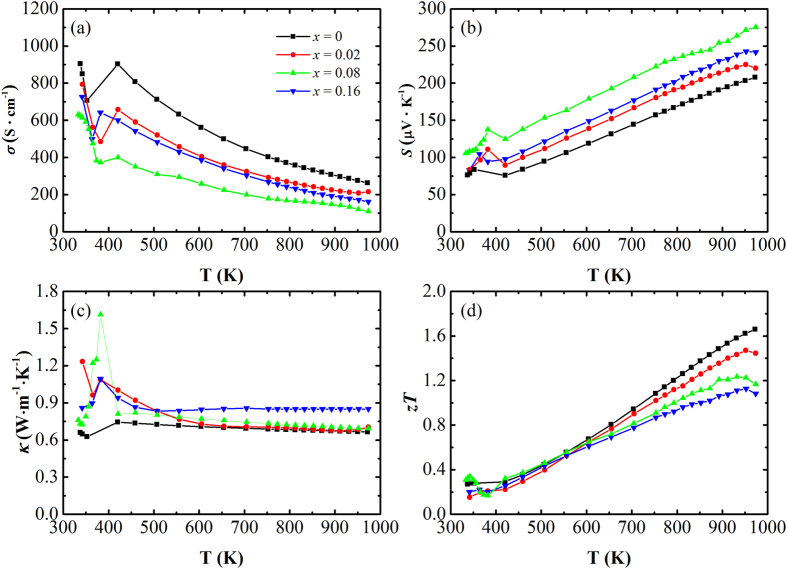
Temperature dependence of thermoelectric properties for the obtained Cu_1.98_S_*x*_Se_1−*x*_ (*x* = 0, 0.02, 0.08, 0.16) bulks: (**a**) electrical conductivity (*σ*), (**b**) Seebeck coefficient (*S*), (**c**) total thermal conductivity (*κ*), and (**d**) dimensionless figure-of-merit (*zT*).

Figure 3a shows the temperature dependence of the electrical conductivity for the Cu_1.98_S_*x*_Se_1−*x*_ (*x* = 0, 0.02, 0.08, 0.16) bulks. It indicates that compared to Cu_1.98_Se, the Cu_1.98_S_*x*_Se_1−*x*_ (*x* = 0.02, 0.08, 0.16) samples have lower *σ* values over the whole measured temperature range, and the most obvious difference occurs at T = 420 K between 400 S · cm^−1^ for the Cu_1.98_S_0.08_Se_0.92_ and 900 S · cm^−1^ for the Cu_1.98_Se. It should be pointed out that this difference becomes less obvious with increasing temperature because the high temperature phases are superionic conductors.

Figure 3b displays the temperature dependence of the Seebeck coefficient for the Cu_1.98_S_*x*_Se_1−*x*_ (*x* = 0, 0.02, 0.08, 0.16) bulks. It reveals that the Cu_1.98_S_*x*_Se_1−*x*_ (*x* = 0.02, 0.08, 0.16) samples have larger *S* values than the Cu_1.98_Se. Specifically, among all the samples, the Cu_1.98_S_0.08_Se_0.92_ has the highest *S* values, around 275 μV · K^−1^ at T = 970 K, which is over 30% higher than that of the Cu_1.98_Se.

Figure 3c shows the temperature dependence of the thermal conductivity for the Cu_1.98_S_*x*_Se_1−*x*_ (*x* = 0, 0.02, 0.08, 0.16) bulks. It indicates that both the Cu_1.98_S_0.02_Se_0.98_ and Cu_1.98_S_0.08_Se_0.92_ bulks have almost the same *κ* values as the Cu_1.98_Se, especially in the temperature range from 500 to 1000 K. The Cu_1.98_S_0.16_Se_0.84_, however, shows increased values over the whole temperature range from 300 to 1000 K.

The temperature dependence of the dimensionless figure-of-merit (*zT*) for the Cu_1.98_S_*x*_Se_1−*x*_ (*x* = 0, 0.02, 0.08, 0.16) bulks is shown in Fig. 3d. It should be noted that the Cu_1.98_S_*x*_Se_1−*x*_ (*x* = 0.02, 0.08, 0.16) samples show almost the same *zT* values as the Cu_1.98_Se in the temperature range from 400 to 600 K. Furthermore, they have *zT* values over 1.0 when T > 800 K and exhibit a peak *zT* at T around 950 K, with the highest value of 1.5 occurring for the Cu_1.98_S_0.02_Se_0.98_.

**Figure 4:**
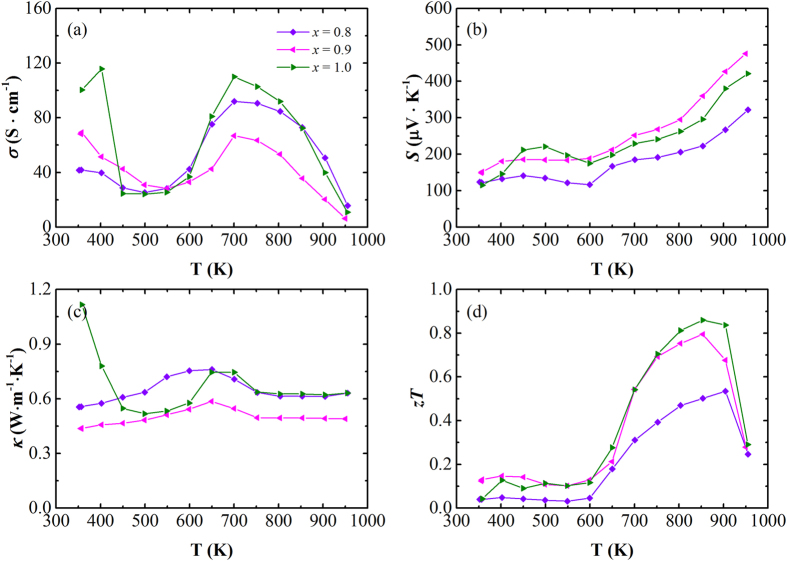
Temperature dependence of the thermoelectric properties for the obtained Cu_1.98_S_*x*_Se_1−*x*_ (*x* = 0.8, 0.9, 1.0) bulks: (**a**) electrical conductivity (*σ*), (**b**) Seebeck coefficient (*S*), (**c**) total thermal conductivity (*κ*), and (**d)** dimensionless figure-of-merit (*zT*).

Figure 4 displays the temperature dependence of the electrical conductivity (a), Seebeck coefficient (b), total thermal conductivity (c), and dimensionless figure-of-merit (d) for the obtained Cu_1.98_S_*x*_Se_1−*x*_ (*x* = 0.8, 0.9, 1.0) bulks. The results indicate that, for the high temperature cubic structured Cu_2_S phase, the Cu_1.98_S_*x*_Se_1−*x*_ samples do not show a monotonic increase or decrease in their electrical conductivity compared to the Cu_1.98_S. The same trend is also observed for the temperature dependence of the Seebeck coefficient. Furthermore, the Cu_1.98_S_0.8_Se_0.2_ sample has almost the same thermal conductivity as the Cu_1.98_S, while the Cu_1.98_S_0.9_Se_0.1_ sample has much smaller thermal conductivity, with values around 0.5 W · m^−1^ · K^−1^ in the temperature range from 700 to 1000 K. This paradoxical result might be related to the characteristics of the superionic system and the larger radius as well as the heavier mass of selenium compared to sulphur. As aforementioned, the cubic structured Cu_2_S is a superionic conductor and it is the migration of copper ions that predominately determines its thermal conductivity. Therefore, the selenium doped samples should have higher thermal conductivity than the Cu_1.98_S, due to the enlarged lattice parameters evidenced from the refinements of the X-ray diffraction patterns. On the other hand, selenium is much heavier than sulphur, so the selenium doped samples should have lower thermal conductivity. As a result of these two opposite factors, the samples show the complicated and varying trend that is observed for the thermal conductivity.

The temperature dependence of the dimensionless figure-of-merit shown in Fig. 4d reveals that the selenium doping does not improve the overall thermoelectric performance of the Cu_1.98_S system, with the Cu_1.98_S having the highest *zT* values around 0.86 at 850 K among all the orthorhombic structured samples, even though certain thermoelectric parameter is enhanced. This observation is in good agreement with our previous reports on the tellurium and iodine doped Cu_2−*x*_Se system^21^, which provides further evidence of the distinctiveness of superionic thermoelectric materials in comparison with the conventional thermoelectric materials.

**Figure 5:**
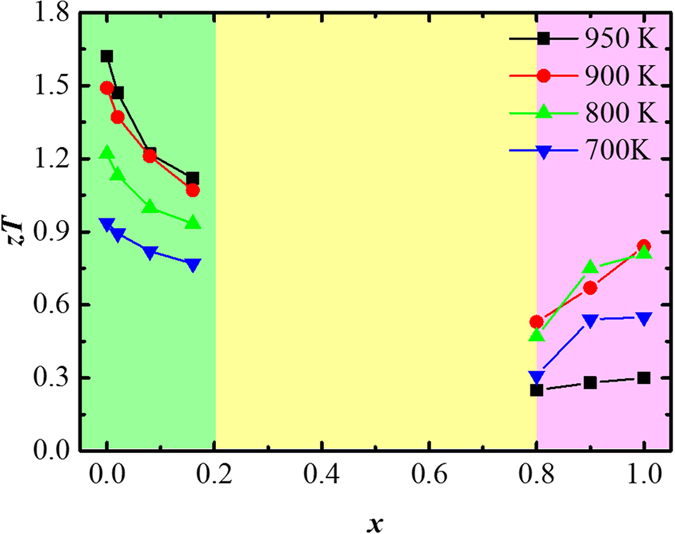
Phase diagram of sulphur doping level dependence of the dimensionless figure-of-merit.

Figure 5 shows a summarized phase diagram for the sulphur doping level dependence of the dimensionless figure-of-merit for the obtained Cu_1.98_S_*x*_Se_1−*x*_ (*x* = 0, 0.02, 0.08, 0.16, 0.8, 0.9, 1.0) samples. It indicates that the *zT* values are first reduced as *x* increases from 0 to 0.16, and then they are enhanced as *x* increases from 0.8 to 1.0, which agrees very well with our previous theoretical and experimental discussions on sulphur (selenium) doping effects on the superionic Cu_2−*x*_Se(S) system.

For the practical applications of thermoelectric materials, besides the high *zT* values that are needed, the thermoelectric compatibility factor, s, is another important factor that needs to be considered when designing an efficient thermoelectric generator^12^,^32^,^35^. The maximum efficiency of a thermoelectric generator will be decreased greatly if the compatibility factors for the segments of *n*- and *p*-type thermoelectric materials differ from each other by a factor larger than two. Therefore, in order to discover the best applicable temperature range and best matching material for a certain thermoelectric material, the calculation of s is quite essential.

**Figure 6:**
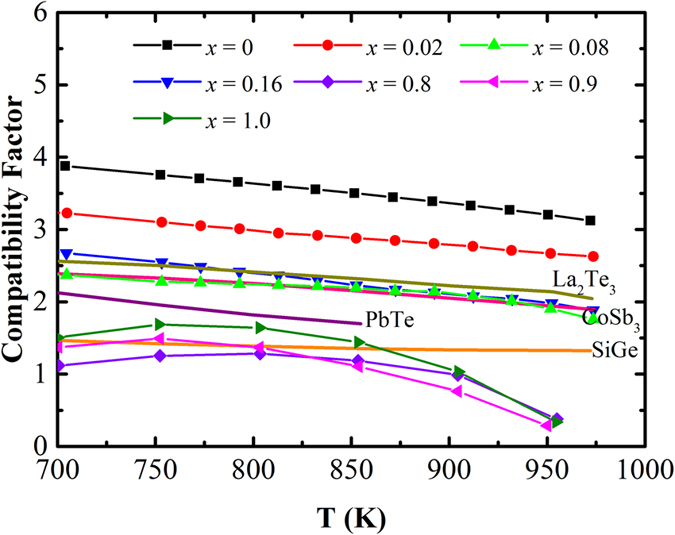
Temperature dependence of the thermoelectric compatibility factor for the Cu_1.98_S_*x*_Se_1−*x*_ (*x* = 0, 0.02, 0.08, 0.16, 0.8, 0.9, 1.0) samples.

Figure 6 shows the temperature dependence of the thermoelectric compatibility factor for the Cu_1.98_S_*x*_Se_1−*x*_ (x = 0, 0.02, 0.08, 0.16, 0.8, 0.9, 1.0) bulks. It indicates that all the samples exhibit stable s values with small fluctuations over a large temperature range from 700 to 1000 K. In the measured temperature range, average s values of around 3.85, 2.92, 2.14, and 2.56 were achieved for the Cu_2_Se-phase Cu_1.98_Se, Cu_1.98_S_0.02_Se_0.98_, Cu_1.9_8S_0.08_Se_0.92_, and Cu_1.98_S_0.16_Se_0.84_ samples, respectively. Furthermore, the Cu_2_S-phase samples Cu_1.98_S_0.8_Se_0.2_, Cu_1.98_S_0.9_Se_0.1_, and Cu_1.98_S exhibit average s values of ~0.72, 0.87, and 0.93 in the temperature range from 700 to 1000 K, respectively.

For comparison purposes, the s values for some well-known n-type thermoelectric materials are also provided in Fig. 6. It indicates that polycrystalline SiGe, PbTe, La_2_Te_3_, and CoSb_3_ bulks show s of around 1.37, 1.90, 2.14, and 2.31, respectively. It should be noted that these are very close to the s values of the Cu_1.98_S_*x*_Se_1−*x*_ (*x* = 0, 0.02, 0.08, 0.16, 0.8, 0.9, 1.0) bulks, with the differences less than a factor of 2 in the temperature range from 700 to 1000 K. Therefore, a relatively high efficiency could be gained from their coupled thermoelectric modules, which is gratifying for their future practical applications.

As aforementioned in the introduction part, the electrical conductivity and Seebeck coefficient is related to the charge carrier density, carrier mobility, and effective mass, which can be deduced from the calculated DOS and electronic band structures. Therefore, in order to fundamentally understand the strange sulphur doping effects on the thermoelectric properties of the Cu_1.98_S_*x*_Se_1−*x*_ system, it is much necessary to do some first-principles calculations on the system’s electronic band structures and DOS.

**Figure 7:**
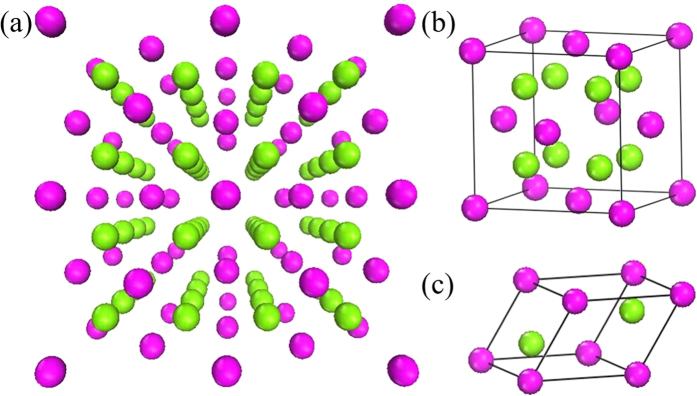
(**a**) Perspective view of the crystal structure for high temperature cubic structured Cu_2_Se(S). (**b**) Ideal version of the unit cell for the cubic structured Cu_2_Se(S). (**c**) Primitive cell for the cubic structured Cu_2_Se(S). Cu and Se(S) atoms are represented by green and purple spheres, respectively.

Figure 7 shows a perspective view of the crystal structures for the cubic structured Cu_2_Se(S), as well as the ideal versions of the unit cell^22^ and the primitive cell. It indicates that, in the ideal version of the unit cell for the cubic structured Cu_2_Se(S) with space group of 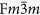, the selenium (sulphur) atoms form a face-centred cubic (*fcc*) sub-lattice, and the copper atoms occupy the tetrahedral interstitial positions. It should, however, be noted that *β*-Cu_2_Se and *α*-Cu_2_S have been reported to be superionic conductors, in which the copper ions behave like a liquid^17^,^19^,^36^,^37^,^38^, and they are kinetically disordered throughout the whole structure.

**Figure 8:**
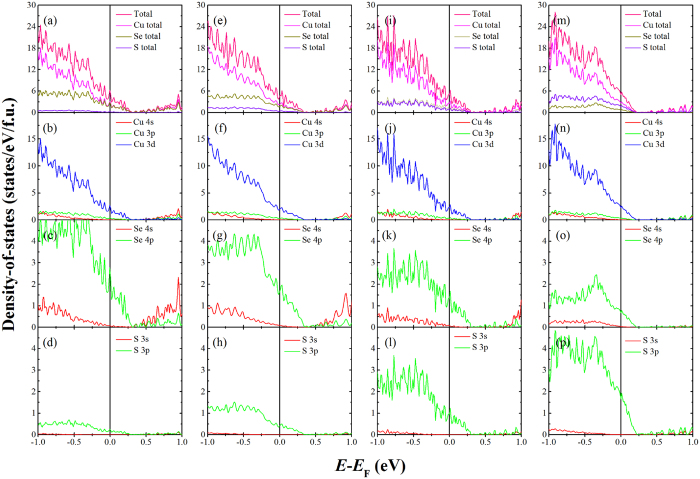
Calculated total and partial density-of-states (DOS) for the Cu_15_S_*x*_Se_8−*x*_ (*x* = 1, 2, 4, 6) compounds obtained from the Density Functional Theory calculations. (**a**,**b**,**c**,**d**) total and partial DOS for the Cu_15_S_1_Se_7_. (**e**,**f**,**g**,**h**) total and partial DOS for the Cu_15_S_2_Se_6_. (**i**,**j**,**k**,**l**) total and partial DOS for the Cu_15_S_4_Se_4_. (**m**,**n**,**o**,**p**) total and partial DOS for the Cu_15_S_6_Se_2_. The total DOS for the Cu, Se, and S atoms are displayed, and the partial DOS for the 4s-, 3p-, and 3d-states of Cu, the 4s- and 4p-states of Se, and the 3s- and 3p-states of S are also presented. The vertical lines mark the position of the Fermi level (*E*_F_).

**Figure 9:**
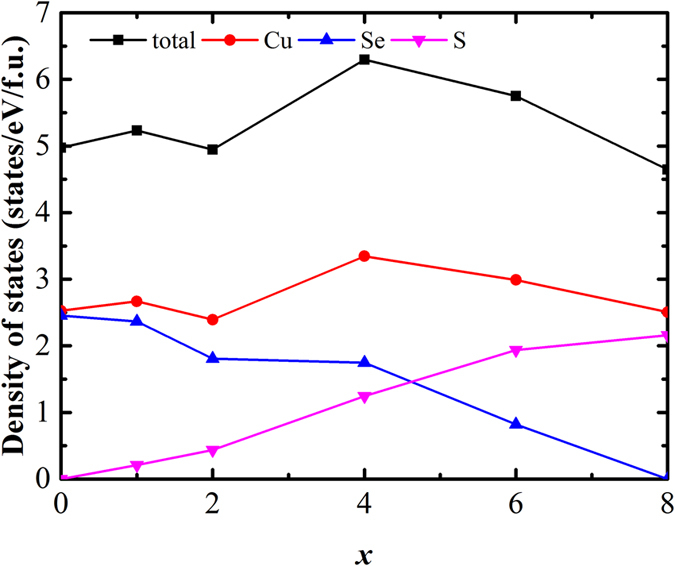
Sulphur doping level dependence of the total and partial density-of-states near the Fermi level for the Cu_15_S_*x*_Se_8−*x*_ (*x* = 0, 1, 2, 4, 6, 8) compounds.

Figure 8 shows the calculated total and partial DOS for the Cu_15_S_*x*_Se_8−*x*_ (*x* = 1, 2, 4, 6) compounds. The sulphur doping level dependence of the total and partial DOS at *E*_F_ for the Cu_15_S_*x*_Se_8−*x*_ (*x* =  0, 1, 2, 4, 6, 8) compounds is displayed in Fig. 9. The results indicate that the DOS at *E*_F_ consists of the contributions from the Cu 3d-states, Se 4p-states, and S 3p-states of the Cu_15_S_*x*_Se_8−*x*_ compounds, with the Cu 3d-states predominately determining the total DOS at *E*_F_. In addition, the partial DOS for the S atoms obviously increases with increasing *x*, while the partial DOS for the Se atoms shows the opposite trend, decreasing with increasing *x*. The total DOS exhibits the same non-monotonic tendency as the partial DOS for the Cu atoms, further indicating that the DOS at *E*_F_ for this system is mainly determined by the copper atoms rather than the S or Se atoms. In summary, the total DOS firstly increases slightly, then clearly decreases, with the Cu_15_S_6_Se_2_ having the highest DOS among all the compounds, ~6.29856 states/eV/f.u.

**Figure 10:**
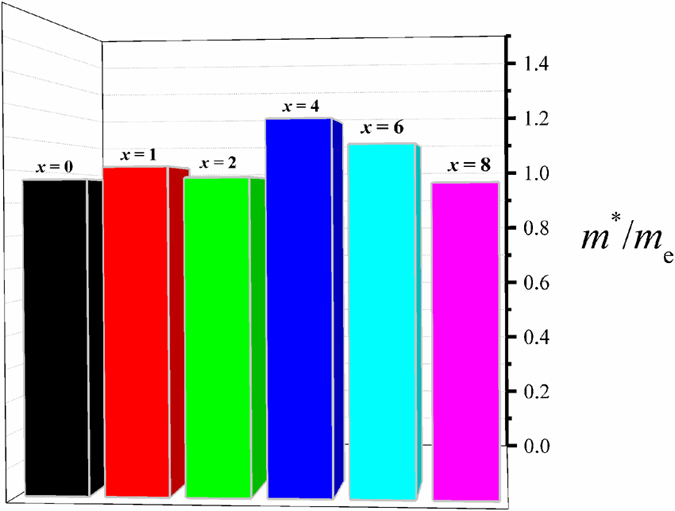
Calculated effective mass for the Cu_15_S_*x*_Se_8−*x*_ (*x* = 0, 1, 2, 4, 6, 8) compounds.

The effective mass can be directly deduced from the calculated DOS based on some references^13^,^39^. Figure 10 shows the deduced effective mass for the Cu_15_S_*x*_Se_8−*x*_ (*x* = 0, 1, 2, 4, 6, 8) compounds. It indicates that, similar to the DOS, the sulphur doping also has a non-monotonic effect on the effective mass. It firstly has positive effects and gives the Cu_15_S_4_Se_4_ its highest m^*^, with a value of ~1.151 *m*_e_. It then shows negative effects and results in decreased m^*^ values as the sulphur doping level increases.

According to Equations 1–4 and previous discussions on *σ, S, n* and *μ*, we know that both *σ* and *S* are linked to the m^*^ and the DOS. Specifically, *σ* is proportional to the carrier density (*n*) and inversely proportional to m^*^. *S*, however, is proportional to m^*^ and 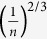. Therefore, based on the obtained information on the DOS and m^*^, we can anticipate the variation trends for the values of *S. σ* values can also be roughly estimated with disregarding the average scattering time. Hence, we can predict that the cubic structured sulphur doped Cu_2−*x*_Se compounds should exhibit non-monotonic variation in *σ* and *S* with increasing sulphur concentration according to the calculated DOS and m^*^ using the DFT method.

It should be pointed out that, for the monoclinic structured Cu_2_Se phased Cu_1.98_S_*x*_Se_1−*x*_ samples, the observed sulphur doping level dependence of the electrical conductivity and the Seebeck coefficient is in good agreement with the theoretical predictions. The DOS is enhanced as the sulphur doping level increases, while the effective mass is reduced as the doping level increases. Additionally, *σ* and *S* are proportional to 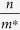 and m^*^, respectively. Therefore, enhanced electrical conductivity and reduced Seebeck coefficient can be obtained when the contribution from the DOS is stronger than that from m^*^. When the contribution of the effective mass is stronger, decreased electrical conductivity and increased Seebeck coefficient will be achieved.

In summary, the Cu_1.98_S_*x*_Se_1−*x*_ compounds have the same crystal structure as monoclinic structured Cu_2_Se when *x* ≤ 0.16, become composites of cubic structured Cu_1.8_Se and hexagonal structured Cu_2.001_S when 0.2 ≤ *x* ≤ 0.7, and finally have the same crystal structure as orthorhombic structured Cu_2_S when 0.8 ≤ *x* ≤ 1.0. The overall thermoelectric performance of the Cu_1.98_S_*x*_Se_1−*x*_ compounds is mainly correlated with the electron effective mass and the density of states, with the *zT* values first increasing and then decreasing. Additionally, all the samples show stable thermoelectric compatibility factors over a broad temperature range from 700 to 1000 K, which could greatly benefit their practical applications. DFT calculations indicate that sulphur doping has non-monotonic effects on the DOS and m^*^, with the Cu_15_S_6_Se_2_ and Cu_15_S_4_Se_4_ having the highest DOS value, ~0.69 states/eV/f.u., and the highest m^*^, ~0.336 *m*_e_, respectively.

## Methods

### Sample preparation

Polycrystalline Cu_1.98_S_*x*_Se_1−*x*_ pellets were synthesized by a conventional solid-state method. Mixtures of Cu, S, and Se powders in the molar ratios of 1.98 : *x* : 1 − *x (x* = 0, 0.02, 0.08, 0.16, 0.2, 0.3, 0.4, 0.5, 0.6, 0.7 0.8, 0.9, 1.0) were pressed into pellets and sealed in evacuated quartz tubes, then heated to 873 K for 1–5 hours with a heating rate of 5 K/min, followed by a furnace cooling to room temperature. Finally, the as-sintered pellets were used in a melt-solidification approach to achieve highly dense polycrystalline bulks, which has been described in detail in our previous work^19^,^21^. The obtained polycrystalline bulks were then shaped into round disks and rectangular bulks for electrical conductivity and thermal diffusivity measurements, respectively.

### Measurements

X-ray diffraction (XRD) patterns were collected on a GBC MMA system using Cu Kα radiation. The electrical conductivity and Seebeck coefficient were measured simultaneously in a helium atmosphere from 300 to 973 K using an RZ2001i system. The thermal diffusivity (D) was measured by the laser flash method (LINSEIS LFA 1000), and the specific heat (*C*_p_) was determined by differential scanning calorimetry (NETZSCH DSC 204F1). The sample density (*dd*) was determined by the sample mass divided by volume, and the thermal conductivity (*κ*) was calculated according to *κ* = D × *C*_p_ × *dd*.

### Calculations

The calculations of electronic band structures, and total and partial DOS were performed based on the DFT method, implemented by the CASTEP package^40^ with the generalized gradient approximation (GGA)^41^. The calculations were parameterized by the Perdew-Burke-Ernzerhof (PBE)^42^ and ultra-soft pseudo-potentials. The plane wave cut-off energy was set at 400 eV. For Cu_2_Se and Cu_2_S, a primitive cell with the Brillouin zone path of ΓXWLΓK (Fig. S1 in Electronic Supplementary Information) was employed for the band structure calculations. For the Cu_15_S_*x*_Se_8−*x*_ (*x* = 0, 1, 2, 4, 6, 8), geometry optimizations were performed on a 2 × 2 × 2 supercell, with one copper atom deleted, of the Cu_2_Se primitive cell. Then, the Brillouin zone path of ΓXWLΓK and a k-point set of 15 × 15 × 15 for the supercell were used to calculate the electronic band structures, and the total and partial DOS, respectively.

## Additional Information

**How to cite this article**: Zhao, L. *et al*. Improvement of thermoelectric properties and their correlations with electron effective mass in Cu_1.98_S_*x*_Se_1−*x*_. *Sci. Rep.* **7**, 40436; doi: 10.1038/srep40436 (2017).

**Publisher's note:** Springer Nature remains neutral with regard to jurisdictional claims in published maps and institutional affiliations.

## Supplementary Material

Supplementary Information
