# Supplementary material for: Improvement of thermoelectric properties and their correlations with electron effective mass in Cu1.98SxSe1−x
Source: Sci Rep. 2017 Jan 16;7:40436. doi: 10.1038/srep40436 (PMC5238374; doi:10.1038/srep40436)
Supplement: Supplementary Information [file srep40436-s1.pdf]

Supplementary Information

Improvement of thermoelectric properties and their correlations with electron effective mass in $Cu_{1.98}S_xSe_{1-x}$

Lanling Zhao[1, 2], Frank Yun Fei[2], Jun Wang[3], Funing Wang[1], Chunlei Wang[1], Jichao Li[1], Jiyang Wang[4], Zhenxiang Cheng[2], Shixue Dou[2], and Xiaolin Wang[2, *]

[1] School of Physics, Shandong University, Jinan, 250100, P.R. China

[2] Spintronic and Electronic Materials Group, Institute for Superconducting and Electronic Materials, Australian Institute for Innovative Materials, University of Wollongong, North Wollongong, 2500, Australia

[3] Key Laboratory for Liquid-Solid Structural Evolution and Processing of Materials, Shandong University, Jinan, 250061, China

[4] Institute for Crystal Materials, Shandong University, Jinan, 250100, P. R. China

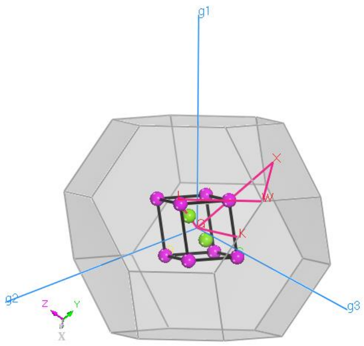

Figure S1. First Brillouin zone of the primitive cell for the cubic structured Cu_2Se. The blue lines indicate the Brillouin zone path ΓXWLΓK used for the electronic band structure calculations.

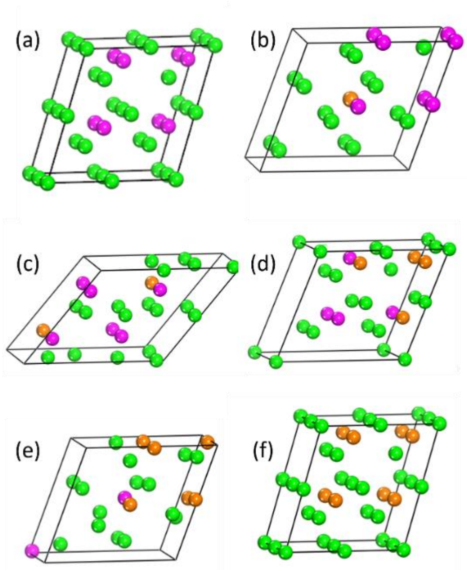

Figure S2 The optimized primitive cell for the copper deficient $Cu_{15}S_xSe_{8-x}$ (x = 0, 1, 2, 4, 6, 8) compounds. (a) $Cu_{15}Se_8$; (b) $Cu_{15}S_1Se_7$; (c) $Cu_{15}S_2Se_6$; (d) $Cu_{15}S_4Se_4$; (e) $Cu_{15}S_6Se_2$; (f) $Cu_{15}S_8$. Cu, Se, and S atoms are represented by green, purple and orange spheres, respectively.

Table S1 Lattice parameters for the geometry optimized primitive cells of the $Cu_{15}S_xSe_{8-x}$ (x = 0, 1, 2, 4, 6, 8) compounds.

Compounds	Space group crystal class	a(Å)	b(Å)	c(Å)	α	β	γ	volume(Å^3)
$Cu_{15}Se_8$	-43m	8.184054	8.184054	8.184054	60	60	60	387.606
$Cu_{15}SSe_7$	3m	8.122183	8.122183	8.122183	59.92842	59.92842	59.92842	378.266
$Cu_{15}S_2Se_6$	mm2	8.066255	8.06625	8.06625	120.21749	119.575	90.1811	371.675
$Cu_{15}S_4Se_4$	-43m	8.00884	8.00884	8.00884	60	60	60	363.241
$Cu_{15}S_6Se_2$	3m	7.87974	7.87974	7.87974	60.8756	60.8756	60.8756	352.784
$Cu_{15}S_8$	-43m	7.83799	7.83799	7.83799	60	60	60	340.486

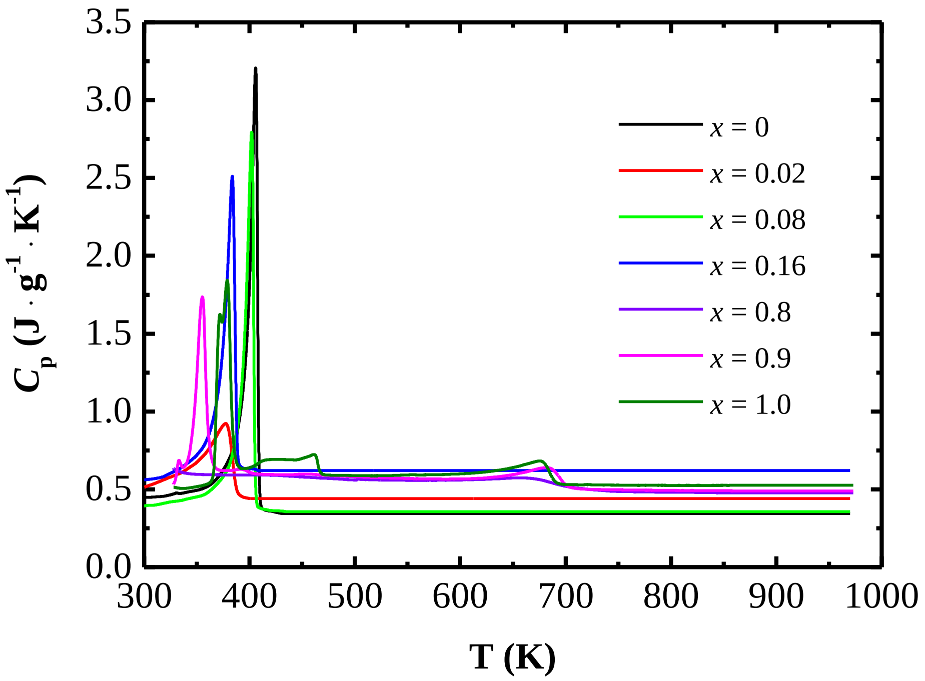

Figure S3 Temperature dependence of specific heat for the obtained $Cu_{1.98}S_xSe_{1-x}$ (x = 0, 0.02, 0.08, 0.16, 0.8, 0.9, 1.0) samples.

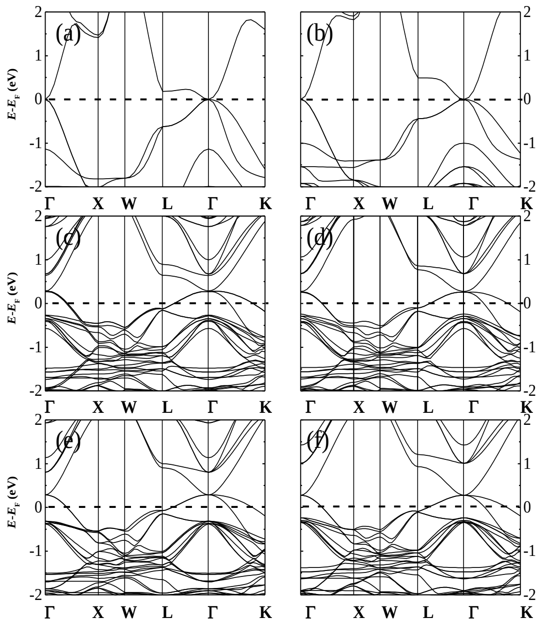

Figure S4 Typical calculated band structures for the Cu_2Se, Cu_2S, and $Cu_{15}S_xSe_{1-x}$ (x = 0, 1, 4, 8) compounds. (a) Calculated band structure for Cu_2Se. (b) Calculated band structure for Cu_2S. (c) Calculated band structure for $Cu_{15}Se_8$. (d) Band structure for the $Cu_{15}S_1Se_7$. (e) Band structure for $Cu_{15}S_4Se_4$. (f) Band structure for $Cu_{15}S_8$.

Table S2 Lattice parameters, and profile (R_p) and weighted profile (R_{wp}) R-factors for the $Cu_{1.98}S_xSe_{1-x}$ (x = 0, 0.02, 0.08, 0.16, 0.2, 0.3, 0.4, 0.5, 0.6, 0.7, 0.8, 0.9, 1.0) samples deduced from Rietveld refinements of the XRD patterns. (Phase 1: cubic structured $Cu_{1.8}Se$, Phase 2: hexagonal structured $Cu_{2.001}S$).

Sample		a (Å)	b (Å)	c (Å)	β (°)	V (Å^3)	R_p	R_{wp}
$Cu_{1.98}Se$		7.117(7)	12.358(6)	27.278(2)	94.112(6)	2393.3(4)	1.820	2.793
$Cu_{1.98}S_{0.02}Se_{0.98}$		7.101(2)	12.301(4)	27.044(0)	94.193(3)	2356.1(0)	3.115	4.536
$Cu_{1.98}S_{0.08}Se_{0.92}$		7.050(5)	12.251(1)	27.237(8)	94.182(7)	2346.4(4)	2.745	3.600
$Cu_{1.98}S_{0.16}Se_{0.84}$		7.072(2)	12.223(9)	27.185(6)	94.186(6)	2343.9(2)	2.556	3.378
$Cu_{1.98}S_{0.2}Se_{0.8}$	Phase 1	5.435(3)	5.435(3)	5.435(3)	90.0(0)	160.5(7)	2.594	3.472
	Phase 2	4.069(1)	4.069(1)	6.891(8)	120.0(0)	98.8(2)		
$Cu_{1.98}S_{0.3}Se_{0.7}$	Phase 1	5.430(2)	5.430(2)	5.430(2)	90.0(0)	160.1(2)	2.917	3.769
	Phase 2	4.056(7)	4.056(7)	6.879(4)	120.0(0)	98.0(5)		
$Cu_{1.98}S_{0.4}Se_{0.6}$	Phase 1	5.415(2)	5.415(2)	5.415(2)	90.0(0)	158.8(0)	2.713	3.687
	Phase 2	4.036(8)	4.036(8)	6.859(9)	120.0(0)	96.8(1)		
$Cu_{1.98}S_{0.5}Se_{0.5}$	Phase 1	5.387(0)	5.387(0)	5.387(0)	90.0(0)	156.3(3)	2.398	3.260
	Phase 2	4.022(7)	4.022(7)	6.846(3)	120.0(0)	95.9(4)		
$Cu_{1.98}S_{0.6}Se_{0.4}$	Phase 1	5.368(8)	5.368(8)	5.368(8)	90.0(0)	154.7(5)	3.493	4.429
	Phase 2	4.003(3)	4.003(3)	6.820(0)	120.0(0)	94.6(6)		
$Cu_{1.98}S_{0.7}Se_{0.3}$	Phase 1	5.390(7)	5.390(7)	5.390(7)	90.0(0)	156.6(5)	2.939	4.243
	Phase 2	3.993(5)	3.993(5)	6.806(2)	120.0(0)	94.0(0)		
$Cu_{1.98}S_{0.8}Se_{0.2}$		15.375(0)	11.954(3)	13.562(1)	116.278(2)	2235.0(7)	1.644	4.779
$Cu_{1.98}S_{0.9}Se_{0.1}$		15.317(9)	11.929(1)	13.536(2)	116.356(1)	2216.3(4)	1.546	2.214
$Cu_{1.98}S$		15.155(7)	11.828(9)	13.401(1)	115.955(7)	2160.15(3)	2.565	3.555
